# Effects of 12 Weeks of Family and Individual Multi-Disciplinary Intervention in Overweight and Obese Adolescents under Cardiometabolic Risk Parameters: A Clinical Trial

**DOI:** 10.3390/ijerph20206954

**Published:** 2023-10-20

**Authors:** Déborah Cristina de Souza Marques, Lilian Rosana dos Santos Moraes, Marilene Ghiraldi de Souza Marques, Joed Jacinto Ryal, Isabella Caroline Santos, Marielle Priscila De Paula Silva Lalucci, Jorge Mota, Pablo Valdés-Badilla, Greice Westphal Nardo, Braulio Henrique Magnani Branco

**Affiliations:** 1 Postgraduate Program in Health Promotion, Cesumar University, Maringá 87050-390, Brazil; deborahmarq@gmail.com (D.C.d.S.M.); lilian.moraes@unicesumar.edu.br (L.R.d.S.M.); marilene.marques@unicesumar.edu.br (M.G.d.S.M.); joed.ryal@unicesumar.edu.br (J.J.R.); mariellepriscila@gmail.com (M.P.D.P.S.L.); braulio.branco@unicesumar.edu.br (B.H.M.B.); 2 Interdisciplinary Laboratory of Intervention in Health Promotion, Cesumar Institute of Science, Maringá 87050-390, Brazil; isabellacaroline_@hotmail.com; 3 Research Centre of Physical Activity, Health, and Leisure, Laboratory for Integrative and Translational Research in Population Health (ITR), Faculty of Sports, University of Porto, 4200-450 Porto, Portugal; jmota@fade.up.pt; 4 Department of Physical Activity Sciences, Faculty of Education Sciences, Universidad Católica del Maule, Talca 3530000, Chile; valdesbadilla@gmail.com; 5 Sports Coach Career, School of Education, Universidad Viña del Mar, Vinã del Mar 2520000, Chile; 6 Medicine Course, Department of Health Sciences, Cesumar University, Maringá 87050-390, Brazil

**Keywords:** multi-professional team, health promotion, adolescent health

## Abstract

Adolescence is a complex period of human development in which young people are susceptible to unhealthy behaviors, such as physical inactivity and an unbalanced diet. This study aimed to analyze the effects of 12 weeks of multi-disciplinary family and individual intervention on cardiometabolic risk parameters in overweight and obese adolescents and compare sub-groups, considering possible differences between sexes (males vs. females vs. intervention approach). Forty-three adolescents (13.73 ± 2.46 years old) of both sexes were divided into two groups: family group (FG) (*n =* 21; 14.24 ± 2.61 years old) and individual group (IG) (*n =* 22; 13.23 ± 2.27 years old). The following parameters were evaluated: anthropometry (body weight, height, waist circumference (WC), hip circumference (HC), abdominal circumference (AC), calculation of body mass index (BMI), and waist–hip ratio (WHR)), body composition (fat mass (FM), lean mass (LM), fat-free mass (FFM), skeletal muscle mass (SMM), body fat percentage (BF), and visceral fat), biochemical measures (fasting glucose, triglycerides (TG), total cholesterol (TC), low-density lipoproteins (LDL-c), and high-density lipoproteins (HDL-c)), and the measurement of systolic and diastolic blood pressure (SBP and DBP) before and after the interventions. The multi-disciplinary interventions occurred for 12 weeks (three days a week lasting 1 h and 30 min, in which 30 min were dedicated to theoretical interventions (nutrition: nutritional education and psychology: psychoeducation) and 1 h to physical exercises. A time effect was observed for LM, FFM, SMM, FM, and HDL-c, with higher values after intervention and a significant decrease for FM, BF, visceral fat, fasting glucose, TG, TC, LDL-c, and DBP (*p <* 0.05). However, no group, sub-group, or interaction effects were observed when comparing FG, IG, or sexes (*p >* 0.05). The responses of the present study show that both multi-disciplinary approaches (family and individual) promoted improvement in the body composition indicators, biochemical markers, and DBP of overweight and obese adolescents independently of the intervention group. Given this finding, health professionals, families, and adolescents could choose the type of intervention based on their preferences.

## 1. Introduction

Weight gain in youth is a global health problem [1]. Adolescents with overweightness or obesity experience emotional, social, and physical health impacts, impairing psychosocial development in this age group [2]. Preventing overweightness, therefore, is a public health priority. If untreated, the excessive load of adiposity is associated with immediate and long-term complications to health at the biopsychosocial level [3,4].

Immediate health risks include changes in systolic and diastolic blood pressure (SBP and DBP), insulin resistance, changes in serum lipoprotein levels (especially biochemical markers associated with increased cardiovascular risk), and triglycerides (TG) [5]. If changes are not prevented, in the long term, childhood obesity persists into adulthood, increasing the risk of the involvement of associated comorbidities, such as type 2 diabetes mellitus, systemic arterial hypertension, dyslipidemia, cancer, and even metabolic syndrome [6].

Therefore, encouraging lifestyle changes by stimulating the consumption of fresh foods and reducing the consumption of ultra-processed foods, in addition to regular physical activity practice, can stop the progression of weight gain and mitigate the consequences of weight gain [7,8,9,10,11]. However, as eating behaviors and body weight are challenging to modify directly, involving family members can help in the process of change and adherence to healthy habits, promoting a non-obesogenic environment to reduce possible health problems [12,13].

Considering the listed aspects, the identification of possible positive outcomes of physiological and metabolic responses in different models of multi-professional intervention, i.e., physical exercise, food re-education, and psychoeducation in family interventions (adolescents and parents receiving multi-professional care together vs. care of only adolescents—individual action), could determine which therapeutic model can be more effective at combatting obesity in adolescence and if there is a better model to be followed for the treatment of this chronic non-communicable disease [14].

Understanding different therapeutic approaches to combat obesity can help health professionals to incorporate more assertive interventional actions into their clinical practices [14]. Therefore, this study aimed to analyze the effects of 12 weeks of family multi-professional intervention (adolescents and guardians) versus isolation (only with adolescents) with overweightness/obesity under cardiometabolic risk parameters, and the second objective was to compare sub-groups by considering possible differences between sex (males vs. females vs. intervention approach). As a hypothesis, it is believed that adolescents who practice multi-professional activities with their parents or guardians may have better results in cardiometabolic risk variables when compared to adolescents who perform the activities proposed alone.

## 2. Materials and Methods

This study presents a randomized clinical trial of parallel groups in two arms, with pre- and post-intervention assessments over 12 weeks of intervention. The adolescents were randomized into two groups (1:1—simple randomization, with subjects being randomly distributed for every assignment, means via http://www.random.com) (accessed on 20 March 2021): a family group (the adolescent participated with the father, mother, or legal guardian) and an individual group (only the adolescent performed the interventions). In addition, for the family group, the same person participated in all interventions, so it was not possible to exchange family members. Prior to the interventions, the adolescents performed the following procedures: (i) medical clearance with information on family history, pre-existing diseases, blood pressure measurement, and pubertal staging via self-reporting; (ii) anthropometry and body composition; and (iii) blood collection for analysis of biomarkers associated with cardiometabolic risk (fasting glucose, triglycerides (TG), total cholesterol (TC), low-density lipoproteins (LDL-c) and high-density lipoproteins (HDL-c)). After the battery of assessments, interventions were initiated.

**Figure 1:**
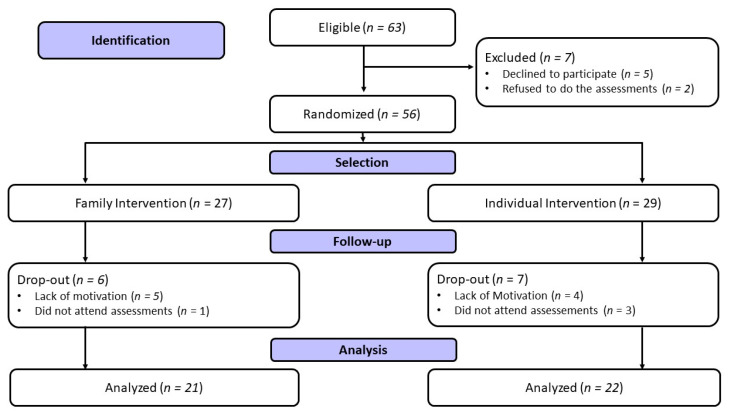
Study flow chart based on CONSORT.

Sixty-three adolescents randomized into two experimental groups were eligible, but 7 dropped out after the beginning of the interventions. In total, 27 adolescents were allocated to the family intervention group and 29 to the individual intervention group (Figure 1). In the end, 43 adolescents were analyzed: 21 adolescents in the family intervention group (male: *n* = 12 and female: *n* = 9) and 22 adolescents in the individual intervention group (male: *n* = 8 and female: *n* = 14) living in a municipality in Southern Brazil. The recruitment of participants was carried out in schools, primary health units, and pediatric clinics through the dissemination of the project through posters and pamphlets, which were also disclosed on the social networks Instagram and Facebook, and advertising on websites, television, and radio programs available in the metropolitan Maringá region.

For this study, selected individuals (i) were between 12 and 17 years old, (ii) were classified as overweight or obese (>85th percentile of body mass index), and (iii) adhered to at least 85% of the requirements in all the multi-professional interventions. Adolescents who (i) presented orthopedic, cardiovascular, or cognitive problems that made it impossible to perform physical exercises, (ii) participated in another multi-disciplinary program or restrictive caloric diet (hypocaloric, low-fat, or low carb), (iii) were using appetite control medications or psychotropic drugs, and (iv) suffered an accident that prevented participation in practical interventions were excluded from this study. According to Jensen et al., 15 adolescents in each intervention group would efficiently verify an α = 0.05 and β = 0.80 [15].

The Local Ethics and Research Committee approved the project under number 4.913.453/2021. Parents or guardians signed the Informed Consent Form, and the adolescents signed the Assent Form. This study followed all the recommendations proposed in Resolution 466/2012 of the Brazilian Ministry of Health. After the approval of the Local Ethics and Research Committee, the project was registered on the Brazilian Clinical Trials Registry Platform (REBEC) under the number RBR-8fp63gm. Figure 1 presents the flow chart of this study according to the Consolidated Standards of Reporting Trials (CONSORT) [16].

Before the beginning of the activities, all adolescents underwent a medical consultation on the premises of the Laboratory of Exercise Physiology of the Educational Institution. In the medical clearance stage, participants were interviewed with a structured anamnesis to investigate their clinical history, socioeconomic status, previous and current pathologies, and use of medicines.

At the beginning and end of the multi-disciplinary interventions, pulmonary and cardiac auscultation were performed, in addition to the measurement of blood pressure (SBP and DBP), according to the recommendations of the 8th Brazilian Guideline of Systemic Arterial Hypertension [17]. SBP and DBP were measured by a highly trained evaluator with an intraclass coefficient (IC) of 0.99 via a manual sphygmomanometer (PREMIUM^®^, São Paulo, Brazil) and stethoscope (LITTMANN^®^ model Classic, New York Mills, MN, USA), with collection performed in the sitting position, after five minutes of rest [17].

The monitoring of pubertal development was performed via the Tanner Scale to systematize the sequence of pubertal events in both sexes by utilizing five stages, considering, in females, sex, breast development, and the distribution and quantity of hair, and in males, the appearance of genital organs and the amount and distribution of pubic hair [18].

The height was measured using the stadiometer brand (Sanny^®^, São Paulo, São Paulo, Brazil) standard, following the standardization proposed by Lohman, Roche, and Martorell [19]. Neck (NC), arm, abdominal (AC), waist (WC), and hip (HC) circumferences (cm) were measured via a flexible tape measure (Cescorf^®^, Porto Alegre, Rio Grande do Sul, Brazil), with a measurement capacity of 2 m and an accuracy of 0.1 cm. Body composition was assessed via the tetrapolar bioimpedance of eight tactile points of the InBody 570^®^ device, through which the following variables were collected: (i) body weight (kg), (ii) fat mass (FM) (in kg and %), (iii) skeletal muscle mass (SMM) (in kg), (iv) fat-free mass (FFM) (in kg), and (v) visceral fat.

For the assessments, adolescents were asked to follow the following guidelines for the examination: (i) fasting from solids and liquids for approximately 12 h, (ii) not using diuretic substances within 24 h of the procedure, (iii) not undertaking moderate or high-intensity physical exercises on the day before the test, (iv) avoiding consuming caffeine-based beverages for 12 h, (v) avoiding the need to urinate or evacuate for 30 min before performing assessments, (vi) not using any metal earrings or accessories at the time of collection; and, finally, (vii) wearing light clothing at the time of assessment [20,21]. With the values found, it was possible to calculate the values of the waist–hip ratio (WHR), the division of WC by HC, and the body mass index (BMI) data, and we classified the nutritional statuses of adolescents according to the cut-off points established by the World Health Organization according to these percentiles: for overweightness, those considered were between the 85th percentile and the <95th, and for obesity, those considered were at the 95th percentile or above [22].

On a pre-determined day and by the scheduled time, the adolescents went to the university laboratory facilities to undergo biochemical analysis in the morning after a night fast of approximately 12 h. After local asepsis of the arm, a puncture was conducted in the antecubital veins of the evaluated patients. All analyses were performed by a biomedical professional, who was shielded to the interventions; the said team did not have access to the intervention model and developed over the 12 weeks. The following biomarkers were analyzed: fasting glucose, TG, TC, LDL-c, and HDL-c. After collection, the samples were centrifuged at 3.600 rpm for 10 min at room temperature (24 °C) to separate serum and plasma. The analyses were performed in triplicate using reagents acquired from Siemens^®^ (Frimley, Camberley, UK) according to the specifications established by the manufacturer. Vacuum tubes (Becton Dickinson—Vacutainer^®^, Plymouth, UK) were used for all collections: the tube with potassium fluoride and ethylenediaminetetraacetic acid (EDTA) for the analysis of fasting glucose (fluoridated plasma) and the tube with clot activator (silica) for the analysis of TC, HDL-c, LDL-c and TG (serum) were used. Siemens equipment (Advia 1800 Chemistry Analyzer^®^, Siemens Healthcare Diagnostics, Tarrytown, NY, USA) was used for biochemical analysis. The classifications of cut-off points of cardiometabolic risk variables were referenced according to the recommendations of the Brazilian Society of Cardiology [23].

The interventions took place over 12 weeks, occurring three days a week (Monday, Wednesday, and Friday). The meetings lasted an hour and a half and included the participation of a team of nutritionists (once a week, every Monday, lasting 30 min), psychologists (once a week, every Wednesday, lasting 30 min), and exercise physiologists (three times a week, lasting 60 min). The interventions were previously prepared and discussed among health professionals to determine a schedule and enable planning between areas. Thus, the contents were elaborated, with common points focusing on behavioral change and the adoption of an active and healthy lifestyle by adolescents over the preceding weeks. In addition, the theoretical and practical interventions of the family intervention were performed with the family, i.e., the guardian together with the child, and the individual, i.e., just the adolescent, participated in the lessons—all the intervention sections occurred in this form.

The nutrition meetings were held in groups, focusing on changing eating behavior. The classes were prepared by nutritionists, with theoretical and practical activities, and always encouraged the development of culinary skills to help adolescents to become independent and gain autonomy when choosing food preferences [14]. Both the family intervention group and the individual intervention group received the same intervention. The meetings were held in groups, focusing on changing eating behavior. The two experimental groups had the same intervention with materials, conversations, and activities that could be carried out with social and group distancing. The intervention lasted approximately 30 min, occurring once a week for 12 consecutive weeks, using the Food Guide for the Brazilian Population as a theoretical reference [24].

These interventions focused on weight loss, improving eating behavior, and empowering healthy eating. As the adolescents performed the bioimpedance test to analyze body composition, the theme was as follows:

(i) The interpretation of the body composition assessment in the electrical bioimpedance: to explain how to read and interpret the result of this test. The adolescents were also encouraged to look at body weight and the quality of body composition, such as FM, BF, SMM, FFM, and visceral fat.
(ii) Pre- and post-workout eating: to demonstrate the importance of diet and its relationship with exercise, examples of foods that can help, the quantities needed, and responses time after consumption were considered.
(iii) Healthy eating: the test discusses the food builders, regulators, and energy of different foods; their due quantities; and their position in the food pyramid. Finally, the researchers explained how to assemble a healthy dish in this class.
(iv) Healthy dish: encouraging adolescents to assemble it and explaining the importance of assembling a suitable dish daily.
(v) The micronutrient class: the importance of vitamins and minerals in adolescent health, nutritional interactions, and examples of the nutrients that we consume in food.
(vi) The fiber class: the importance of consuming fiber daily, the amount required, the difference between soluble and insoluble fibers, and where to find the different fibers present in food.
(vii) Food labels: how to read food labels, as well as practical examples, such as juices from sachets, biscuits, and processed foods.
(viii) Food labels in “food for children and adolescents”: to understand kilocalories, macronutrients, micronutrients, preservatives, stabilizers, and dyes in “common” industrialized food.”
(ix) Physical or emotional hunger: explain in detail how to identify the hunger level and whether it is physical or emotional.
(x) Review of class content: to observe if the children and adolescents understand the topics addressed so far, such as (a) how many types of fiber there are, (b) how to assemble a healthy dish, etc.
(xi) Myths and truths of nutrition: to explain some myths commonly commented on between this age, such as “water fasting with slimming lemon”, "sweating makes you lose weight”, “do not eat carbohydrates to lose weight”, etc.
(xii) Final lesson: recommending how to behave on vacation (without the research group) and the ten steps to encourage healthy eating.

Concerning the nutritional data of this study, the results have already been published in another study that discussed food-level processing [14].

Group psychoeducation was also carried out through theoretical and practical activities. Parents or guardians and teenagers were encouraged to participate in the conversations. The central issues were elaborated based on the National Health Promotion Policy (NHPP) [24] to reduce symptoms and disorders via cognitive behavioral strategies [9,25]. Both the family and individual groups performed interventions once a week, totaling 12 meetings. Each meeting lasted 30 min. Group psychoeducation was achieved through theoretical and practical activities, in which parents and adolescents participated in conversations and activities that led to reflection on biopsychosocial health. At the end of the sessions, participants received explanatory folders on controlling anxiety and binge eating and strategies to promote “mindfulness”. The activities were based on operative groups [26]. Thus, the group approach provided the exchange in knowledge and stimulus to critical thinking to help adolescents understand how to think, feel, and act in the face of possible obesogenic environments. Psychoeducation classes followed the topics below:

(i) The intention was to meet the participants in the project and understand their expectations for the next 12 weeks, as well as inform them how the meetings would occur and what would be developed.
(ii) Health and mental health: adolescents were encouraged to understand what health is and its benefits and assist themselves in understanding the environment and context concerning their senses and emotions.
(iii) Self-image: The identification of the individual by himself can be used to consider elements of the external world, such as parental figures, friends, idols, or even cultural characteristics. Thus, understanding themselves and their reality can help them to control their emotions and behaviors; therefore, a framework was developed for the participants to fill in to explain how they feel about the contexts inserted, such as school, project, work, and home.
(iv) Sleep: a theoretical class to gain an understanding of sleep via an illustrative presentation of these concepts in a participatory model; the goal was to promote a perception of its importance and how it affects their health, as well as to stimulate self-analysis of this aspect in their lives.
(v) Anxiety: theoretical–practical classes provided dynamically and playfully identification of the physical and psychological sensations of anxiety and caused self-perception of these since the theme was about anxiety.
(vi) Pathological anxiety: a theoretical class was provided on the differentiation between normal and pathological anxiety to reflect that, in some cases, it is a natural response of the human being.
(vii) Self-control: following the knowledge previously acquired about anxiety, a more theoretical practical class was held, outlining techniques to assist in anxious moments or crises to promote bodily, psychological, spatial, and temporal self-awareness.
(viii) Communication in the family environment: this class was more theoretical in terms of the relationship between parents and children, but in conversation, the format explained how to improve the quality of the relationship and communication between parents and children and how to deal with daily challenges.
(ix) Emotional intelligence: A theoretical class was used to provoke a self-analysis and discussion about how feelings and communication affect relationships with peers and oneself. For this class, the dynamic proposed was the “Bingo in Emotions,” where the individual drew emotions, and the emotion drawn should be manifested to explain how to act and deal with it in the context into which it was inserted.
(x) Self-sabotage: to lead participants to reflect and self-perceive how their emotions and beliefs can affect the slimming process, promoting self-sabotage.
(xi) Leisure: to ensure that the participants understood the importance of leisure for mental health and their family relationships, as well as how it can increase the lengths of their lives.
(xii) A recap of the content taught throughout the 12 weeks: we recapitulated on all the themes and encouraged adolescents to follow up after completing the multi-disciplinary project.

The training sessions were developed in a circuit format, divided into two mesocycles (each with a six-week duration), with training sessions A and B (divided into exercises with body weight, appliances, and accessories, prioritizing the large muscle groups) shown in Table 1 and Table 2. The physical exercises did not have a repetition count, but the active and passive time of the exercises was controlled via the effort and pause ratio. In the first mesocycle, the effort:pause ratio was 30″ by 30″, whereas in the second mesocycle, the effort ratio:pause was 40″ by 20″, using a 1:1 ratio between the concentric and eccentric phases. An anatomical adaptation was initially performed in the first weeks to plan training sessions and reduce possible injuries through a gradual increase in effort intensity via the Foster scale [27].

Table 1 and Table 2 present the physical exercises (training sessions A and B) for 12 weeks of intervention.

After confirming the normality of the data via the Kolmogorov–Smirnov test, the data were presented as the mean and standard deviation, relative values (%), and the calculation of the absolute delta (∆ = post- minus pre-intervention values). Regarding inferential statistics, a two-way analysis of variance (ANOVA) was performed to compare possible intra-group and inter-group differences (possible effects of group, time, and interaction) and apply the Bonferroni post hoc test if a significant difference was detected in ANOVA. For all analyses, a *p* < 0.05 was assumed. In addition, another secondary analysis was conducted; an ANOVA was then applied, separating the groups (adolescents and parents or guardians and adolescents alone) by sex, and pre- and post-intervention comparisons were performed between groups and sexes. As a final point, the effect size was calculated using Cohen’s d [28]: 0.20 to 0.49 (small effect), 0.50 to 0.79 (moderate effect), and >0.80 (large effect). The effect size for eta square (ŋ²) was calculated according to Richardson et al. [28]: 0.0099 (small effect), 0.0588 (moderate effect), and 0.1379 (large effect). All statistical analyses were performed using the statistical package Statistica (Version 12.0, Stasoft, United States of America).

## 3. Results

In the present study, 43 adolescents (23 girls and 20 boys) were evaluated, of whom 21 (9 girls and 12 boys) participants were allocated to the family group, and 22 (14 girls and 8 boys) were assigned to the individual group. There was a significant difference for pubertal staging, with an increase in the stage of sexual maturation in the reassessments after 12 weeks of intervention (*p =* 0.03). However, this result was already expected, given that adolescents are in a period of growth and body complexion. Table 3 presents information about anamnesis and the general characteristics of both intervention groups.

Among the drugs used, the adolescents’ responses were as follows: class of hormone replacement drugs, anticholinergics, antihypertensives, antidiabetics, antidepressants, retinoids, and amphetamines. Table 4 presents the measurements of sample characteristics before and after multi-professional intervention in terms of group and sex comparisons.

For age, a time effect was only detected in the comparisons between the two experimental groups (F_1,41_ = 14.22; *p* = 0.0005; Ƞ² = 0.25; large effect), with the Bonferroni post hoc showing higher values for the post-intervention (*p* < 0.0001), as well as for comparison between the groups separated by sex (F_1,39_ = 11.71; *p* = 0.001; Ƞ² = 0.23; large effect), with the Bonferroni post hoc showing higher values for the post-intervention (*p* = 0.0006).

For height, a time effect was only observed in the comparison between the two experimental groups (F_1,41_ = 14.96; *p* = 0.012; Ƞ² = 0.26; large effect), with the Bonferroni post hoc showing higher values after the intervention period (*p* = 0.0003), as well as for comparison between groups separated by sex (F_1,39_ = 15.16; *p* = 0.0003; Ƞ² = 0.28; large effect), with the Bonferroni post hoc showing higher values for the post-intervention (*p* = 0.0003).

For body weight, BMI, BMI z-score, and SBP, no group, time, and interaction effects were also identified for the two intervention groups and comparison between the groups separated by sex (*p* > 0.05).

For DBP, there was only a time effect (F_1,41_ = 7.58; *p* = 0.008; Ƞ² = 0.16; large effect), with the Bonferroni post hoc detecting lower values after the intervention period (*p* = 0.008). Table 5 shows the adolescents’ anthropometric parameters and body composition before and after the interventions.

For LM, a time effect was only observed in the comparison between the two experimental groups (F_1,41_ = 22.68; *p* < 0.001; Ƞ² = 0.36; large effect), with the Bonferroni post hoc showing higher values after the intervention period (*p* < 0.001), and the same was observed for the sub-comparisons by sex, that is, we only observed a time effect (F_1,39_ = 20.55; *p* < 0.0001; Ƞ² = 0.34; large effect), with the Bonferroni post hoc showing higher values for the post-intervention time (*p* < 0.0001).

For the FFM, there was also only a time effect for comparison between the two experimental groups (F_1,41_ = 24.15; *p* < 0.0001; Ƞ² = 0.37; large effect), with the Bonferroni post hoc indicating higher values after the intervention period (*p* < 0.0001), as was also true for the sub-comparations by sex (F_1,39_ = 21.82; *p* < 0.0001; Ƞ² = 0.35; large effect), with the Bonferroni post hoc showing higher values for the post-intervention time (*p* < 0.0001).

For the SMM, a time effect was only observed in the comparison between the two experimental groups (F_1,41_ = 26.98; *p* < 0.0001; Ƞ² = 0.39; large effect), with the Bonferroni post hoc showing higher values after the intervention period (*p* < 0.0001), and the same trend was observed for the sub-comparations by sex (F_1,39_ = 24.71; *p* < 0.0001; Ƞ² = 0.38; large effect), with the Bonferroni post hoc showing higher values for the post-intervention time (*p* < 0.0001).

For FM, there was also only a time effect for comparison between the two experimental groups (F_1,41_ = 12.26; *p* = 0.001; Ƞ² = 0.23; large effect), with the Bonferroni post hoc presenting lower values after the intervention period (*p* = 0.001), as was true for the sub-comparations between the sexes (F_1,39_ = 11.13; *p* < 0.001; Ƞ² = 0.22; large effect), with the Bonferroni post hoc showing lower values for the post-intervention time (*p* < 0.001).

For the BF, there was only a time effect for the comparison between groups (F_1,41_ = 22.29; *p* < 0.001; Ƞ² = 0.35; large effect), with the Bonferroni post hoc indicating lower values after the intervention period (*p* < 0.001), as well as for the sub-comparations by sex (F_1,39_ = 21.06; *p* < 0.001; Ƞ² = 0.35; large effect), with the Bonferroni post hoc showing lower values for the post-intervention time (*p* < 0.001).

For the level of visceral fat, there was also only a time effect for comparisons between groups (F_1,41_ = 7.17; *p* = 0.01; Ƞ² = 0.15; large effect), with the Bonferroni post hoc indicating lower values after the intervention (*p* = 0.010), as well as sub-comparations between sexes (F_1,39_ = 7.54; *p* = 0.009; Ƞ² = 0.16; large effect), with the Bonferroni post hoc showing lower values for the post-intervention time (*p* = 0.01). For WC, AC, HC, and WHR, there were no effects of group, time, or interaction (*p* > 0.05).

Table 6 shows the biochemical responses of the two experimental groups stratified by sex and in general terms before and after the interventions.

For fasting glucose, there was only a time effect in the comparison between groups (F_1,41_ = 13.81; *p* = 0.0006; Ƞ² = 0.25; large effect), with the Bonferroni test showing lower values after the intervention period (*p* = 0.0006). However, for the sub-comparison between sexes, a group effect was observed (F_3,39_ = 3.74; *p* = 0.01; Ƞ² = 0.22; large effect), with the Bonferroni post hoc showing higher values for the group (boys—individual intervention) when compared to the group (girls—individual intervention) (*p* = 0.03), along with a time effect (also for the sub-group) (F_3,39_ = 12.24; *p* = 0.001; Ƞ² = 0.23; large effect), with higher values at the pre-intervention time when compared to the post-intervention (*p* = 0.0008).

For TC, there was only a time effect in the comparison between groups (F_1,41_ = 18.98; *p* = 0.0008; Ƞ² = 0.32; large effect), with the Bonferroni post hoc indicating lower values after the intervention period (*p* = 0.0008), meaning that the same occurred for the sub-comparisons by sex, that is, only a time effect was detected (F_1,39_ = 19.09; *p* = 0.00008; Ƞ² = 0.32; large effect), with the Bonferroni post hoc showing lower values for the post-intervention (*p* = 0.0005).

For HDL-c, there was only a time effect in the comparison between groups (F_1,41_ = 62.43; *p* < 0.0001; Ƞ² = 0.60; large effect), with the Bonferroni post hoc indicating higher values after the intervention period (*p* < 0.0001), as well as for the sub-comparison between sex (F_1,39_ = 55.30; *p* < 0.0001; Ƞ² = 0.58; large effect), with the Bonferroni test showing higher values for the post-intervention (*p* < 0.0001).

For LDL-c, there was only a time effect in the comparison between groups (F_1,41_ = 154.97; *p* < 0.0001; Ƞ² = 0.79; large effect), with the Bonferroni test showing lower values after the intervention period (*p* < 0.0001), and the same trend was observed for the sub-comparison between sex (F_1,39_ = 154.72; *p* < 0.00001; Ƞ² = 0.79; large effect), with the Bonferroni post hoc showing lower values for the post-intervention (*p* < 0.0001).

For TG, there was only a time effect in the comparison between groups (F_1,41_ = 8.74; *p* = 0.005; Ƞ² = 0.17; large effect), with the Bonferroni post hoc indicating lower values after the intervention period (*p* = 0.004), and the same trend occurred for the sub-comparisons by sex (F_1,39_ = 6.98; p = 0.01; Ƞ² = 0.15; large effect), with the Bonferroni test showing higher values for the post-intervention (*p* = 0.005).

## 4. Discussion

Considering that this study aimed to analyze the effects of 12 weeks of family and individual multi-disciplinary intervention in overweight and obese adolescents under cardiometabolic risk parameters, the main results of the present study after the intervention process were as follows:

(i) A decrease in visceral fat, FM, BF, fasting glucose, TC, LDL-c, and TG for both groups, regardless of the type of intervention;
(ii) An increase in LM, FFM, SMM, and HDL-c, regardless of the type of intervention;
(iii) A significant reduction in DBP in the two intervention groups.

On the other hand, no significant differences were observed for the other variables analyzed in the present study or for comparisons by sex, i.e., male vs. female (the unique difference observed was fasting glucose between the sub-groups, i.e., male vs. female in pre-intervention time). This study’s hypothesis was not confirmed by the responses observed, since no differences were detected between the two intervention groups’ comparisons.

### 4.1. Anthropometric and Body Composition Responses

Both groups showed favorable changes in body composition parameters and biochemical markers related to cardiometabolic risk, regardless of the intervention group (family vs. individual approach). Given this outcome, it is considered that the best model to be incorporated for health promotion in adolescents with overweightness or obesity will need to be based on the personal choices of adolescents to carry out multi-disciplinary activities (nutrition education, psychoeducational classes, and physical exercises) accompanied by parents or alone. However, it is impossible to state that one model is more effective than another within the age group and level of pubertal development because pre-adolescents may depend more on parents or guardians, whereas adolescents in advanced periods of pubertal development could be more independent in their tasks or daily activities. Obesity and severe obesity cases were reduced by around 9% for all intervention groups post-intervention, which was very relevant to health status [6,10] (conforming to Supplementary Table S1).

Regardless of the absence of differences between the intervention groups, there was an improvement in body composition components, with a reduction in absolute, relative, and visceral fat and an increase in LM, FFM, and SMM. Previous evidence shows that the interventional period analyzed may promote improvements in body composition components in adolescents with overweightness or obesity [7,8,9]. The reduction in adiposity indicators (FM, BF, and visceral fat) is related to a negative energy balance, higher energy expenditure, and lower caloric consumption [29,30], associated with the food education classes and increased level of supervised physical activity in the interventions. In addition, the increase in LM, FFM, and SMM is associated with the stimulus provided by competing exercises: untrained individuals can bring positive physiological adjustments to muscle hypertrophy and cardiorespiratory fitness [31]. The concatenated responses to muscle hypertrophy suggest that the volume and intensity used in training sessions were adequate to increase LM, FFM, and SMM after the intervention period.

No significant differences were observed for anthropometric parameters, i.e., BMI, WC, AC, HC, and WHR. Considering WC, it is observed that as the results presented significant variability (∆ = −7.32 ± 17.63 cm), the factor that may justify the absence of significant differences for WC, although a moderate effect size was observed (d = −0.68; moderate effect) for girls, a relevant finding in the fight against central obesity. Similar responses were found by Branco et al. in interventions with the same duration and a similar methodological approach. Considering that WC is strongly related to cardiometabolic risk [32,33], longer interventions are recommended (>12 weeks) to evaluate anthropometric responses due to a possible reduction in WC, HC, and WHR [7].

Therefore, regardless of significant changes in anthropometric parameters, such as body weight and circumferences, the active involvement of the child/adolescent and the whole family in multi-disciplinary interventions must promote changes in healthy habits and adherence to the intervention process so that the benefits can last into adulthood. Previous studies, with only eight weeks of intervention, comparing interventions for behavior change based on family and interventions only for adolescents also found no significant changes to the BMI z-score [34,35]. In this context, new studies can test similar approaches with longer intervention times to identify possible differences in anthropometric and body composition parameters.

### 4.2. Biomarkers Responses

It is well established in the scientific literature that physical exercise promotes greater glycemic control via an increased sensitivity to insulin action [36]. The sub-group of adolescents who performed the activities without parents/guardians presented values for fasting glucose pre-intervention above the recommended cut-off points (pre: 105.38 ± 7.37 mg/dL), characterized as “pre-diabetes” [36], and 12 weeks of multi-disciplinary intervention were able to reduce fasting glucose (∆ = −4.00 ± 8.59%; with d = −0.54; moderate effect), although the mean values remained slightly above the recommended cut-off point (101.38 ± 10.34 mg/dL) [36]. The other three experimental sub-groups (girls with parents, boys with parents, and girls alone) showed glycemic values within the normality pattern, and the highest fasting blood glucose in the male sub-group without parental/guardian monitoring may be related to the randomization process (since the different values were related to the pre-intervention time). Considering that resistance to insulin action and type 2 diabetes mellitus has affected a higher portion of the young population, it becomes indispensable to carry out interventions based on changes in lifestyle, with weight loss, improved eating patterns (reduction in the consumption of processed and ultra-processed foods), and regular and systematic physical activity practice all important factors [37].

Early identification of parameters related to lipid profile (TC and fractions: LDL-c, VLDL-c, and HDL-c, in addition to triglycerides) can substantially reduce possible cardiovascular risk, and interventions based on lifestyle changes (physical activity and food re-education) promote significant improvements in adolescents with obesity-associated comorbidities [38]. The recent systematic review by Rai et al. (2023) [39] points out that six weeks of physical exercise cause a reduction in triglycerides and increase HDL-c, regardless of the intensity performed [39]. Therefore, in this aspect, the most relevant point is the increase in energy expenditure, regardless of the method or intensity of effort performed. The oxidation process of fatty acids occurs in the active muscle groups (via the bloodstream) [40]. On the other hand, it is discussed that the reduction in TC and LDL-c are related to the type of physical exercise performed, that is, the concurrent training associated with improved lipid profile indicators, especially in pediatric obesity [41]. Independently of the type or intensity of physical activity performed, it is suggested that adolescents perform an average of 60 min of physical activity at moderate to vigorous intensity, mainly via aerobic activities, throughout the week, performing these activities at least 3 times a week [5], to promote better health and quality of life.

As a final point, there was over a 70% reduction in fasting glucose for both intervention groups. Similarly, more than 20% of the adolescents with pre-diabetes returned to normal ranges. For TG, 95% of the sample was in the borderline classification, but after the intervention, these values were reduced by around ~40%. For TC, there was a reduction higher than 20% for borderline and higher classification values post-intervention for both experimental groups. For HDL-c, values increased by approximately ~40% after intervention for all groups. LDL-c returned to average values in ~60% of adolescents after intervention in the two intervention groups (conforming to Supplementary Table S1).

### 4.3. Blood Pressure Responses

A recent meta-analysis of randomized controlled trials showed that interventions lasting more than two weeks may significantly improve blood pressure control, reducing SBP and DBP [42]. In this sense, the responses of SBP and DBP tend to decrease (−6.04/−2.54 mmHg) after concurrent training. In the present study, reductions in SBP delta (∆ = −0.10 ± 10.40 mmHg) were observed in the family intervention group (∆ = −3.18 ± 15.24 mmHg), albeit without a statistical difference; on the other hand, the DBP delta in the family intervention (∆ = −3.81 ± 9.73 mmHg) and individual (∆ = −4.09 ± 9.08 mmHg) groups showed a significant reduction, with lower values recorded after 12 weeks of intervention. The decrease in blood pressure found in this study is associated with the decline in peripheral vascular resistance, resting heart rate (although this measure was not performed in the present study), double product, and arterial stiffness—factors related to post-exercise hypotension [43]. Lastly, there was a relative reduction in high hypertension values for SBP (13 to 17%) and DBP (23 to 31%) for all intervention groups (conforming to Supplementary Table S1).

### 4.4. Health Promotion Considerations

The primary approach must include lifestyle changes, such as increased physical activity levels, reduced screen time, and food re-education, to reduce risks attributed to obesity and associated comorbidities [38,44]. Therefore, it is believed that educational actions are indispensable to empower adolescents to engage in self-care related to physical activity and the consumption of healthy foods. Although the family intervention did not show significantly higher responses than individual intervention, it is recommended that the family is involved in the behavior change process since this support may provide better outcomes in terms of the health components of at-risk adolescents [45].

### 4.5. Limitations, Strengths, and Future Study Possibilities

As limitations of this study, the following points are listed: (i) family involvement and the relationship between adolescents and their responsible adults are factors that can influence the parameters analyzed in this study; (ii) this study was conducted during the COVID-19 pandemic, in which period the world population showed changes in the level of physical activity, school activities (remote classes), and the availability of food (increased consumption of processed and ultra-processed foods); and (iii) a lack of long-term follow-up assessments to verify the behavior and possible differences between the different interventions can reduce the results’ reliability. Strong points are highlighted: (i) the proposal of a low-cost group intervention model that can be implemented in different places and environments, such as schools, community centers, sports centers, basic health units, and hospitals, and (ii) the incorporation of a multi-disciplinary model of primary health care, since obesity has multifactorial causes. Future studies could consider the long-term monitoring responses between the two types of interventions and analyze possible differences between growth stages of development to conduct more assertive interventions for overweight and obese pre-adolescents or adolescents, whether with parents or alone.

## 5. Conclusions

Based on the responses to the present study, it is verified that multi-disciplinary interventions effectively improved body composition components, with a reduction in FM, BF, and visceral fat, in addition to the increase in FFM, LM, and SMM, in the two intervention groups. Moreover, both groups showed a significant reduction in fasting blood glucose, TC, LDL-c, and TG and increased HDL-c. A decrease in DBP was also observed for all experimental groups and sub-groups after 12 weeks of intervention. In clinical practice, the choice of the type of intervention to be performed will depend on the preference of the adolescent, whether performing actions of behavior change with parents or separately. It should be emphasized to parents/guardians that participating in thetheoretical–practical actions developed in this study will not only bring benefits regarding the process of nutritional education, mental health, and the stimulation of physical activity but also provide a favorable environment for behavior change to last, avoiding possible relapses.

## Figures and Tables

**Table 1:** Systematization of concurrent training A during 12 weeks of intervention.

Order	Exercise (s)	Serie (s)	Repetition (s)	Execution Speed
1	Warming up—walking, interval, running	8′	***	1:1
2	Step (up and down)	3x	30″	1:1
2	Naval rope	3x	30″	1:1
3	Standing row with squat	3x	30″	1:1
4	Jump trampoline	3x	30″	1:1
5	Hip bridge	3x	30″	1:1
6	Frontal displacement up to the cone with a jump at the end	3x	30″	1:1
7	Squat with biceps curl	3x	30″	1:1
8	Ladder of agility (front)	3x	30″	1:1
9	Rectus abdominals with lying hip adduction	3x	30″	1:1
10	Dislocation with Mini band	3x	30″	1:1
11	Cool-down period	2′	***	1:1
Stretching				

Note: *** = without repetition. ′ = minutes. ″ = seconds.

**Table 2:** Systematization of concurrent training B during 12 weeks of intervention.

Order	Exercise (s)	Serie (s)	Repetition (s)	Execution Speed
1	Warming up—walking, interval, running	8′	***	1:1
2	Step sideways (Up and down, on one side and the other)	3x	30″	1:1
3	Traction with elastic band	3x	30″	1:1
4	Push-ups	3x	30″	1:1
5	Squat with a ball throw downwards	3x	30″	1:1
6	Agility ladder (side)	3x	30″	1:1
7	Infra-abdominal (feet holding the ball)	3x	30″	1:1
8	Thruster with dumbbells	3x	30″	1:1
9	Zig-zag offset between discs	3x	30″	1:1
10	Jump trampoline	3x	30″	1:1
11	Adapted burpee	3x	30″	1:1
12	Cool-down period	2′	***	1:1
Stretching				

Note: *** = without repetition. ′ = minutes. ″ = seconds.

**Table 3:** General characteristics of the adolescents participating in the present study.

Variables		General (F and M)		Female		Male	
		Family Intervention (*n =* 21)	Individual Intervention (*n =* 22)	Family Intervention (*n =* 9)	Individual Intervention (*n =* 14)	Family Intervention (*n =* 12)	Individual Intervention (*n =* 8)
Health Plan	Yes	5 (23.80%)	5 (22.72%)	6 (66.66%)	5 (35.71%)	3 (25.00%)	0 (0%)
	No	12 (57.14%)	17 (77.27%)	3 (33.33%)	9 (64.28%)	9 (75.00%)	8 (100%)
Educational Level	Primary School	12 (57.14%)	16 (72.72%)	5 (55.55%)	12 (85.71%)	7 (58.33%)	4 (50.00%)
	High School	9 (42.85%)	6 (27.27%)	4 (44.44%)	2 (14.28%)	5 (41.66%)	4 (50.00%)
Income	Low	7 (33.33%)	3 (13.63%)	2 (22.22%)	6 (42.85%)	5 (41.66%)	5 (62.50%)
	Average	9 (42.85%)	8 (36.36%)	4 (44.44%)	5 (35.71%)	5 (41.66%)	3 (37.50%)
	High	5 (23.80%)	11 (50%)	3 (33.33%)	3 (21.42%)	2 (16.66%)	0 (0%)
Medication	Yes	2 (9.52%)	4 (18.18%)	1 (11.11%)	3 (21.42%)	1 (8.33%)	1 (12.50%)
	No	19 (90.47%)	18 (81.81%)	8 (88.88%)	11 (78.57%)	11(91.66%)	7 (87.50%)
Smoker	Yes	0 (0%)	0 (0%)	0 (0%)	0 (0%)	0(0%)	0 (0%)
	No	21 (100%)	22 (100%)	9 (100%)	14 (100%)	12 (100%)	8 (100%)
Alcoholic	Yes	0 (0%)	0 (0%)	0 (0%)	0 (0%)	0 (0%)	0 (0%)
	No	21 (100%)	22 (100%)	9 (100%)	14 (100%)	12 (100%)	8 (100%)

Note: Data are expressed as absolute and relative (%) values; F = female; M = male; low income = 1 to 3 times the minimum wage; average income = 3 to 6 times the minimum wage; high income = 6 to 9 times the minimum wages.

**Table 4:** Measurements of sample characteristics before and after multi-professional intervention in terms of group and sex comparisons.

Variables		Family Intervention (*n* = 21)		∆	Cohen’s *d*	Individual Intervention (*n* = 22)		∆	Cohen’s *d*
		Pre-Intervention	Post-Intervention			Pre-Intervention	Post-Intervention		
Age (years old) *	G F M	14.24 ± 2.61 14.78 ± 2.39 13.83 ± 2.79	14.52 ± 2.62 15.00 ± 2.50 14.17 ± 2.76	0.29 ± 0.46 0.22 ± 2.50 0.33 ± 0.49	0.11 0.09 0.12	13.23 ± 2.27 12.79 ± 2.22 14.00 ± 2.27	13.45 ± 2.39 13.07 ± 2.37 14.13 ± 2.42	0.23 ± 0.43 0.29 ± 0.47 0.13 ± 0.35	0.10 0.13 0.06
Body weight (kg)	G F M	76.95 ± 22.30 67.13 ± 13.35 84.31 ± 25.24	77.47 ± 21.79 67.73 ± 12.45 84.77 ± 24.79	0.52 ± 2.12 0.60 ± 2.13 0.46 ± 2.20	0.02 0.04 0.02	83.52 ± 28.76 85.61 ± 30.64 75.15 ± 17.11	83.22 ± 26.60 85.35 ± 28.61 75.53 ± 16.18	−0.30 ± 3.01 −0.26 ± 3.03 0.38 ± 1.74	−0.01 −0.01 0.07
Height (m²) *	G F M	1.62 ± 0.13 1.56 ± 0.03 1.67 ± 0.16	1.63 ± 0.13 1.57 ± 0.03 1.68 ± 0.16	0.01 ± 0.01 0.00 ± 0.01 0.01 ± 0.02	0.07 0.11 0.08	1.64 ± 0.10 1.62 ± 0.08 1.67 ± 0.14	1.65 ± 0.10 1.63 ± 0.08 1.68 ± 0.12	0.01 ± 0.01 0.00 ± 0.01 0.01 ± 0.02	0.06 0.06 0.07
BMI (kg/m²)	G F M	28.89 ± 6.11 27.43 ± 5.43 29.98 ± 6.59	28.77 ± 5.78 27.54 ± 5.00 29.66 ± 6.31	−0.12 ± 0.98 0.11 ± 0.80 −0.32 ± 1.12	−0.02 0.02 −0.05	30.77 ± 9.47 32.30 ± 10.58 26.59 ± 3.78	30.52 ± 8.84 32.09 ± 9.86 26.50 ± 3.86	−0.25 ± 1.11 −0.21 ± 1.15 −0.09 ± 0.77	−0.03 −0.02 −0.02
BMI score Z	G F M	2.17 ± 1.36 1.54 ± 1.11 2.41 ± 1.99	2.12 ± 1.23 1.60 ± 1.03 2.36 ± 1.81	−0.05 ± 0.26 0.05 ± 0.16 −0.05 ± 0.30	−0.04 0.05 −0.02	2.52 ± 1.63 2.15 ± 1.40 1.68 ± 0.73	2.45 ± 1.53 2.11 ± 1.29 1.65 ± 0.75	−0.07 ± 0.20 −0.04 ± 0.29 −0.04 ± 0.19	−0.05 −0.03 −0.05
SBP (mmHg)	G F M	119.05 ± 11.36 118.89 ± 6.01 120.71 ± 9.97	118.95 ± 6.09 118.89 ± 14.53 119.29 ±13.85	−0.10 ± 10.40 0.00 ± 12.25 −1.43 ± 15.12	0.02 0.00 −0.14	122.73 ± 13.86 119.00 ± 6.41 128.75 ± 12.46	119.55 ± 8.99 119.17 ± 9.00 117.50 ± 7.07	−3.18 ± 15.24 0.17 ± 9.36 −11.25 ± 12.46	0.35 0.03 −1.59
DBP (mmHg) *	G F M	80.00 ± 10.49 83.33 ± 13.23 77.50 ± 7.54	76.19 ± 6.69 78.89 ± 6.01 74.17 ± 6.69	−3.81 ± 9.73 −4.44 ± 10.14 −3.33 ± 9.85	−0.57 −0.74 −0.50	83.64 ± 8.48 82.86 ± 9.14 85.00 ± 7.56	79.55 ± 5.75 80.00 ± 6.79 78.75 ± 3.54	−4.09 ± 9.08 −2.86 ± 9.14 −6.25 ± 9.16	−0.71 −0.42 −1.77

Note: Data are expressed as mean and ± = standard deviation; ∆ = delta (post minus pre-intervention); G = general (female and male group together); F = female; M = male; BMI = body mass index; SBP = systolic blood pressure. DBP = diastolic blood pressure; * = time difference between pre- and post-intervention (*p* < 0.0001).

**Table 5:** Anthropometry and body composition measurements before and after multi-disciplinary intervention in terms of group and sex comparisons.

Anthropometry and Body Composition		Family Intervention (*n* = 21)		∆	Cohen’s *d*	Individual Intervention (*n* = 22)		∆	Cohen’s *d*
		Pre-Intervention	Post-Intervention			Pre-Intervention	Post-Intervention		
WC (cm)	G FM	85.08 ± 12.84 80.14 ± 10.78 89.50 ± 18.72	82.44 ± 19.45 72.82 ± 17.90 89.60 ± 17.84	−2.64 ± 12.87 −7.32 ± 17.63 0.10 ± 3.15	−0.21 −0.68 0.01	88.92 ± 17.62 88.78 ± 13.43 87.90 ± 16.72	88.57 ± 16.27 89.65 ± 17.96 86.76 ± 14.04	−0.35 ± 3.20 0.88 ± 6.60 −1.14 ± 3.36	−0.02 0.07 −0.07
AC (cm)	G FM	93.32 ± 14.90 86.70 ± 10.21 98.29 ± 16.27	91.64 ± 14.84 84.79 ± 9.86 96.78 ± 16.20	−1.68 ± 5.21 −1.91 ± 3.40 −1.51 ± 6.39	−0.11 −0.19 −0.09	97.24 ± 18.56 98.14 ± 20.03 95.66 ± 16.85	97.11 ± 17.91 98.12 ± 19.86 95.35 ± 14.99	−0.12 ± 4.95 −0.01 ± 5.39 −0.31 ± 4.42	−0.01 0.001 −0.02
HC (cm)	G FM	106.12 ± 13.60 103.44 ± 11.80 110.16 ± 17.78	105.10 ± 12.68 101.52 ± 8.05 110.39 ± 16.30	−1.02 ± 4.27 −1.92 ± 6.21 0.23 ± 3.03	−0.07 −0.16 0.01	108.27 ± 17.10 108.13 ± 14.98 104.96 ± 16.45	107.45 ± 16.64 107.78 ± 15.07 102.33 ± 17.05	−0.81 ± 3.43 −0.34 ± 1.97 −2.64 ± 3.49	−0.05 −0.02 −0.16
WHR (cm)	G FM	0.80 ± 0.05 0.77 ± 0.05 0.82 ± 0.04	0.78 ± 0.13 0.72 ± 0.17 0.83 ± 0.08	−0.02 ± 0.12 −0.06 ± 0.16 0.01 ± 0.06	−0.37 −1.03 0.18	0.82 ± 0.06 0.81 ± 0.06 0.84 ± 0.07	0.82 ± 0.07 0.81 ± 0.05 0.85 ± 0.08	0.01 ± 0.04 0.01 ± 0.03 0.02 ± 0.05	0.08 −0.02 0.25
LM (kg) *	G FM	42.87 ± 10.54 36.54 ± 3.89 47.62 ± 11.56	44.22 ± 10.45 37.59 ± 3.66 49.19 ± 11.23	1.35 ± 1.44 1.01 ± 1.49 1.58 ± 1.42	0.13 0.27 0.14	44.73 ± 11.35 42.83 ± 9.88 48.05 ± 13.62	45.57 ± 10.85 43.62 ± 9.65 48.99 ± 12.63	0.85 ± 1.58 0.79 ± 1.18 0.94 ± 2.20	0.07 0.08 0.07
FFM (kg) *	G FM	45.52 ± 11.22 38.83 ± 4.04 50.53 ± 12.37	47.00 ± 11.15 39.94 ± 3.79 52.28 ± 12.02	1.48 ± 1.54 1.11 ± 1.59 1.75 ± 1.50	0.13 0.28 0.14	47.54 ± 11.97 45.51 ± 10.34 51.10 ± 14.45	48.47 ± 11.46 46.39 ± 10.12 52.11 ± 13.42	0.93 ± 1.67 0.89 ± 1.29 1.01 ± 2.29	0.08 0.09 0.07
SMM (kg) *	G FM	24.95 ± 6.59 21.00 ± 2.47 24.77 ± 6.18	25.87 ± 6.57 21.70 ± 2.36 25.34 ± 6.06	0.91 ± 0.89 0.70 ± 0.90 0.56 ± 0.78	0.14 0.28 0.09	26.06 ± 7.21 27.92 ± 7.22 28.31 ± 8.71	26.67 ± 6.92 28.99 ± 7.04 29.01 ± 8.10	0.61 ± 1.03 1.08 ± 0.89 0.70 ± 1.42	0.09 0.15 0.08
FM (kg) *	G FM	31.43 ± 13.29 28.30 ± 9.96 40.10 ± 21.36	30.49 ± 12.96 27.79 ± 9.81 38.96 ± 19.79	−0.94 ± 1.50 −0.51 ± 0.85 −1.14 ± 2.63	−0.07 −0.05 −0.05	35.98 ± 20.70 33.78 ± 15.33 28.78 ± 18.57	34.75 ± 19.31 32.51 ± 15.00 27.38 ± 17.12	−1.24 ± 2.44 −1.27 ± 1.82 −1.40 ± 2.23	−0.06 −0.08 −0.08
BF (%) *	G FM	39.70 ± 8.12 41.03 ± 6.94 43.89 ± 10.23	38.20 ± 8.25 39.94 ± 7.58 42.83 ± 10.10	−1.50 ± 1.83 −1.09 ± 0.95 −1.06 ± 1.88	−0.19 −0.16 −0.10	40.50 ± 11.46 38.70 ± 9.08 34.56 ± 11.67	39.25 ± 11.52 36.88 ± 8.80 32.98 ± 11.76	−1.25 ± 1.99 −1.82 ± 2.28 −1.59 ± 2.27	−0.11 −0.20 −0.14
Visceral Fat (%) *	G FM	14.33 ± 5.48 14.00 ± 4.97 15.43 ± 5.65	13.81 ± 5.88 13.44 ± 5.27 15.21 ± 5.67	−0.52 ± 0.93 −0.56 ± 0.53 −0.21 ± 1.37	−0.10 −0.11 −0.04	14.00 ± 5.81 14.58 ± 6.04 11.50 ± 5.53	13.64 ± 6.03 14.08 ± 6.52 10.88 ± 5.96	−0.36 ± 1.22 −0.50 ± 1.17 −0.63 ± 0.92	−0.06 −0.08 −0.11

Note: data are expressed by mean and ± = standard deviation; ∆ = delta (post minus pre-intervention); G = general (female and male group together); ∆ = delta (post minus pre-intervention); F = female; M = male; WC = waist circumference; AC = abdominal circumference; HC = hip circumference; WHR = waist–hip ratio; LM = lean mass; FFM = fat-free mass; SMM = skeletal muscle mass; LM = lean mass; BF = body fat percentage; * = time difference with pre- and post-intervention (*p* < 0.0001).

**Table 6:** Biochemical responses of the two experimental groups before and after the intervention period in terms of group and sex comparisons.

Variables		Family Intervention (*n* = 21)		∆	Cohen’s *d*	Individual Intervention (*n* = 22)		∆	Cohen’s *d*
		Pre-Intervention	Post-Intervention			Pre-Intervention	Post-Intervention		
Fasting glucose (mg/dL) *	G F M	99.71 ± 9.24 96.76 ± 8.84 101.93 ± 9.27	94.76 ± 10.21 91.56 ± 11.84 97.17 ± 8.54	−4.95 ± 9.26 −5.20 ± 12.11 −4.76 ± 7.02	−0.54 −0.59 −0.51	99.64 ± 8.02 96.36 ± 6.54 105.38 ± 7.37 †	94.95 ± 9.33 91.29 ± 6.58 101.38 ± 10.34	−4.68 ± 7.69 −5.07 ± 7.44 −4.00 ± 8.59	−0.58 −0.78 −0.54
Total cholesterol (mg/dL) *	G F M	174.05 ± 33.41 174.00 ± 35.33 174.04 ± 33.48	158.27 ± 36.45 169.53 ± 36.55 149.82 ± 35.53	−15.78 ± 27.13 −4.47 ± 23.99 −24.27 ± 27.14	−0.47 −0.13 −0.72	174.82 ± 30.64 165.43 ± 30.76 191.25 ± 23.97	155.27 ± 23.18 150.00 ± 22.55 164.50 ± 22.70	−19.55 ± 26.03 −15.43 ± 29.51 −26.75 ± 17.93	−0.64 −0.50 −1.12
HDL-c (mg/dL) *	G F M	37.13 ± 3.85 38.67 ± 4.61 35.98 ± 2.84	46.33 8.02 48.06 ± 6.81 45.03 ± 8.89	9.20 ± 6.44 9.39 ± 6.83 9.06 ± 6.44	2.39 2.04 3.19	38.20 ± 6.13 37.37 ± 4.25 39.66 ± 8.68	46.21 ± 8.74 46.37 ± 9.19 45.94 ± 8.48	8.01 ± 7.75 9.00 ± 6.49 6.28 ± 9.82	1.31 2.12 0.76
LDL-c (mg/dL) *	G F M	134.20 ± 30.06 133.67 ± 29.19 134.60 ± 31.99	95.65 ± 32.01 105.13 ± 27.71 88.54 ± 34.30	−38.55 ± 22.77 −28.54 ± 15.71 −46.06 ± 24.89	−1.28 −0.98 −1.44	133.68 ± 26.92 127.69 ± 28.12 144.18 ± 22.55	92.35 ± 21.40 88.81 ± 24.01 98.53 ± 15.32	−41.34 ± 19.24 −38.87 ± 18.88 −45.65 ± 20.37	−1.54 −1.38 −2.02
Triglycerides (mg/dL) *	G F M	98.29 ± 51.15 86.89 ± 28.69 106.83 ± 63.02	89.90 ± 49.77 80.89 ± 27.41 96.67 ± 61.97	−8.38 ± 28.63 −6.00 ± 12.56 −10.17 ± 36.98	−0.16 −0.21 −0.16	94.82 ± 44.75 103.93 ± 49.50 78.88 ± 31.62	81.86 ± 43.79 87.71 ± 47.60 71.63 ± 36.81	−12.95 ± 17.64 −16.21 ± 17.21 −7.25 ± 18.02	−0.29 −0.33 −0.23

Note: data are expressed as mean and ± = standard deviation; ∆ = delta (post- minus pre-intervention); G = general (female and male group); F = female; M = male; HDL-c = high-intensity lipoproteins; LDL-c = low-intensity lipoproteins; * = time difference between pre- and post-intervention (*p* < 0.0001); † = higher values when compared to the female sub-group without parental or guardian follow-up (*p <* 0.05).

## Data Availability

The data generated during the study will be informed when requested.

## Supplementary Materials

The following supporting information can be downloaded at: https://www.mdpi.com/article/10.3390/ijerph20206954/s1. Table S1: Biochemical, anthropometric, and blood pressure changes per adolescent and classifications before and after the intervention.
