# Supplementary material for: Effects of 12 Weeks of Family and Individual Multi-Disciplinary Intervention in Overweight and Obese Adolescents under Cardiometabolic Risk Parameters: A Clinical Trial
Source: Int J Environ Res Public Health. 2023 Oct 20;20(20):6954. doi: 10.3390/ijerph20206954 (PMC10606176; doi:10.3390/ijerph20206954)
Supplement: Supplementary file 1 [file ijerph-20-06954-s001.zip › ijerph-2625424-supplementary.pdf]

Supplementary Table S1. Biochemical, anthropometric, and blood pressure changes per adolescent and classifications before and after the intervention.

Variables and classification		General (male and female)				Female				Male			
		Family (*n* = 21)		Individual (*n* = 22)		Family (*n* = 9)		Individual (*n* = 14)		Family (*n* = 12)		Individual (*n* = 8)	
Biochemical	Classification	Pre	Post	Pre	Post	Pre	Post	Pre	Post	Pre	Post	Pre	Post
Fasting glucose	Non-diabetics	10 (47.61%)	15 (71.43%)	10 (45.45%)	17 (77.27%)	5 (55.55%)	7 (77.77%)	9 (64.28%)	14 (100.00%)	5 (41.66%)	8 (66.66%)	1 (12.50%)	3 (37.50%)
	Pre-diabetic	11 (52.38%)	6 (28.57%)	12 (54.54%)	5 (22.72%)	4 (44.44%)	2 (22.22%)	5 (35.71%)	0 (0%)	7 (58.33%)	4 (33.33%)	7 (87.50%)	5 (62.50%)
TG	Normal pattern	1 (4.76%)	12 (57.14%)	1 (4.54%)	10 (45.45%)	1 (11.11%)	5 (55.55%)	0 (0%)	6 (42.85%)	0 (0%)	7 (58.33%)	1 (12.50%)	4 (50.00%)
	Borderline	20 (95.23%)	9 (42.85%)	21 (95.45%)	12 (54.54%)	8 (88.88%)	4 (44.44%)	14 (100.00%)	8 (57.14%)	12 (100.00%)	5 (41.66%)	7 (87.50%)	4 (50.00%)
TC	Normal pattern	12 (57.14%)	16 (76.19%)	14 (63.63%)	17 (77.27%)	6 (66.66%)	7 (77.77%)	8 (57.14%)	10 (71.42%)	6 (50.00%)	9 (75.00%)	6 (75.00%)	7 (87.50%)
	Borderline	5 (23.80%)	3 (14.28%)	4 (18.18%)	2 (9.09%)	2 (22.22%)	2 (22.22%)	3 (21.42%)	2 (14.28%)	3 (25.00%)	1 (8.33%)	1 (12.50%)	0 (0%)
	Higher values	4 (19.04%)	2 (9.52%)	4 (18.18%)	3 (13.63%)	1 (11.11%)	0 (0%)	3 (21.42%)	2 (14.28%)	3 (25.00%)	2 (16.66%)	1 (12.50%)	1 (12.50%)
HDL-c	Normal pattern	1 (4.76%)	12 (57.14%)	1 (4.54%)	10 (45.45%)	1 (11.11%)	5 (55.55%)	0 (0%)	6 (42.85%)	0 (0%)	7 (58.33%)	1 (12.50%)	4 (50.00%)
	Below normal	20 (95.23%)	9 (42.85%)	21 (95.45%)	12 (54.54%)	8 (88.88%)	4 (44.44%)	14 (100.00%)	8 (57.14%)	12 (100.00%)	5 (41.66%)	7 (87.50%)	4 (50.00%)
LDL-c	Normal pattern	4 (19.04%)	13 (61.90%)	8 (36.36%)	16 (72.72%)	1 (11.11%)	4 (44.44%)	3 (21.42%)	10 (71.42%)	3 (25.00%)	9 (75.00%)	5 (62.50%)	6 (75.00%)
	Borderline	5 (23.80%)	5 (23.80%)	6 (27.27%)	5 (22.72%)	2 (22.22%)	3 (33.33%)	3 (21.42%)	4 (28.57%)	3 (25.00%)	2 (16.66%)	3 (37.50%)	1 (12.50%)
	Higher values	12 (57.14%)	3 (14.28%)	8 (36.36%)	1 (4.54%)	6 (66.66%)	2 (22.22%)	8 (57.14%)	0 (0%)	6 (50.00%)	1 (8.33%)	0 (0%)	1 (12.50%)
BMI	Overweight	10 (47.63%)	12 (57.15%)	11 (50.01%)	13 (59.10%)	6 (66.66%)	7 (77.77%)	4 (36.36%)	5 (41.66%)	4 (8.33%)	5 (41.66%)	6 (75.00%)	7 (87.5%)
	Obesity	8 (38.09%)	7 (33.33%)	3 (13.63%)	4 (18.18%)	3 (33.33%)	2 (22.22%)	5 (45.45%)	5 (41.66%)	5 (41.66%)	5 (41.66%)	1 (12.50%)	0 (0%)
	Severe Obesity	3 (14.28%)	2 (9.52%)	8 (36.36%)	5 (22.72%)	0 (0%)	0 (0%)	3 (27.27%)	2 (16.68%)	3 (25.00%)	2 (16.66%)	1 (12.50%)	1 (12.50%)
SBP	Normal pattern	12 (57.14%)	16 (76.19%)	14 (63.63%)	17 (77.27%)	6 (66.66%)	7 (77.77%)	8 (57.14%)	10 (71.42%)	6 (50.00%)	9 (75.00%)	6 (75.00%)	7 (87.50%)
	High values	5 (23.80%)	3 (14.28%)	4 (18.18%)	2 (9.09%)	2 (22.22%)	2 (22.22%)	3 (21.42%)	2 (14.28%)	3 (25.00%)	1 (8.33%)	1 (12.50%)	0 (0%)
	Hypertension	4 (19.04%)	2 (9.52%)	4 (18.18%)	3 (13.63%)	1 (11.11%)	0 (0%)	3 (21.42%)	2 (14.28%)	3 (25.00%)	2 (16.66%)	1 (12.50%)	1 (12.50%)
DPB	Normal pattern	10 (47.61%)	15 (71.43%)	10 (45.45%)	17 (77.27%)	5 (55.55%)	7 (77.77%)	9 (64.28%)	14 (100.00%)	5 (41.66%)	8 (66.66%)	1 (12.50%)	3 (37.50%)
	High values	11 (52.38%)	6 (28.57%)	12 (54.54%)	5 (22.72%)	4 (44.44%)	2 (22.22%)	5 (35.71%)	0 (0%)	7 (58.33%)	4 (33.33%)	7 (87.50%)	5 (62.50%)
	Hypertension	0 (0%)	0 (0%)	0 (0%)	0 (0%)	0 (0%)	0 (0%)	0 (0%)	0 (0%)	0 (0%)	0 (0%)	0 (0%)	0 (0%)

Note: Data are expressed as absolute and relative (%) values.; TG = triglycerides (mg/dL); TC = total cholesterol (mg/dL); HDL-c = high-density lipoprotein (mg/dL); LDL-c = low-density lipoprotein (mg/dL); BMI = body mass index (kg/m²); SBP = systolic blood pressure (mmHg) and DBP = diastolic blood pressure (mmHg).
